# *Clostridioides difficile* Infection among Cirrhotic Patients with Variceal Bleeding

**DOI:** 10.3390/antibiotics10060731

**Published:** 2021-06-17

**Authors:** Mirela Nicoleta Voicu, Florica Popescu, Dan Nicolae Florescu, Ion Rogoveanu, Adina Turcu-Stiolica, Dan Ionut Gheonea, Vlad Florin Iovanescu, Sevastita Iordache, Sergiu Marian Cazacu, Bogdan Silviu Ungureanu

**Affiliations:** 1 Department of Pharmacology, University of Medicine and Pharmacy of Craiova, 200349 Craiova, Romania; nicoletamirelavoicu@gmail.com (M.N.V.); prof_floricapopescu@hotmail.com (F.P.); 2 Department of Gastroenterology, University of Medicine and Pharmacy of Craiova, 200349 Craiova, Romania; dan.florescu@umfcv.ro (D.N.F.); ion.rogoveanu@umfcv.ro (I.R.); dan.gheonea@umfcv.ro (D.I.G.); vlad.iovanescu@umfcv.ro (V.F.I.); sevastita.iordache@umfcv.ro (S.I.); sergiu.cazacu@umfcv.ro (S.M.C.); bogdan.ungureanu@umfcv.ro (B.S.U.); 3 Department of Pharmacoeconomics, University of Medicine and Pharmacy of Craiova, 200349 Craiova, Romania

**Keywords:** *Clostridioides difficile*, risk factors, Charlson comorbidity index, Child–Pugh score

## Abstract

*Clostridioides difficile* infection (CDI) stands as the leading cause of nosocomial infection with high morbidity and mortality rates, causing a major burden on the healthcare system. Driven by antibiotics, it usually affects older patients with chronic disease or immunosuppressed or oncologic management. Variceal bleeding secondary to cirrhosis requires antibiotics to prevent bacterial translocation, and thus patients become susceptible to CDI. We aimed to investigate the risk factors for CDI in cirrhotic patients with variceal bleeding following ceftriaxone and the mortality risk in this patient’s population. We retrospectively screened 367 cirrhotic patients with variceal bleeding, from which 25 patients were confirmed with CDI, from 1 January 2017 to 31 December 2019. We found MELD to be the only multivariate predictor for mortality (odds ratio, OR = 1.281, 95% confidence interval, CI: 0.098–1.643, *p* = 0.042). A model of four predictors (age, days of admission, Charlson index, Child–Pugh score) was generated (area under the receiver operating characteristics curve, AUC = 0.840, 95% CI: 0.758–0.921, *p* < 0.0001) to assess the risk of CDI exposure. Determining the probability of getting CDI for cirrhotic patients with variceal bleeding could be a tool for doctors in taking decisions, which could be integrated in sustainable public health programs.

## 1. Introduction

*Clostridioides difficile* infection (CDI) represents the leading cause of nosocomial infection and is associated with high morbidity and mortality as well as increased healthcare costs [1]. A European multicentre study involving 482 hospitals showed a rate of seven cases per 10,000 patients and also revealed a suboptimal laboratory diagnosis in the eastern European countries [2]. While it seems that CDI is shifting towards community spreading, there are still many hospital-acquired cases, especially in patients with comorbidities and if antibiotics are used.

Chronic disease, older age, immunosuppressive and oncologic medication are considered risk factors for patients acquiring hospital CDI [3]. Additionally, the increased use of proton pump inhibitors (PPI) [4] and nonsteroidal anti-inflammatory drugs (NSAID) [5] has been associated with an increase in CDI. Even though any antibiotic may be incriminated for inducing hospital-based infection by disrupting the gut barrier, clindamycin, cephalosporins and fluoroquinolones have been frequently linked to CDI [6]. Liver cirrhosis is the 13th cause of death worldwide, even though the viral aetiology seems to be diminishing [7]. When portal hypertension is present, oesophageal varices will appear and approximately 20% will bleed in the first three years from diagnosis. Despite standard endoscopic therapy for oesophageal varices, some patients will rebleed and other complications may occur either due to portal hypertension or liver insufficiency [8].

Cirrhotic patients are vulnerable to developing CDI infection due to their frequent admission and infections, as well as dysbiosis and a low immune system [9]. Bacterial infection is frequently encountered in cirrhotic patients with variceal bleeding. Both the American Association for the Study of Liver Diseases (AASLD) and the European Association for the Study of the Liver (EASL) recommends the use of ceftriaxone as a prophylaxis for bacterial infection, rebleeding and reduced hospital stay [10,11]. Nonetheless, patients admitted with variceal bleeding have a higher mortality rate and are usually present in an advanced cirrhotic stage with additional possible complications. While the use of ceftriaxone is recommended for 5–7 days, this time, it might be enough for patients to acquire CDI depending on their status.

Additional methods might be required to prevent widespread CDI incidence in cirrhotic patients. Both local and global strategies should ensure new public health actions to consider sustainability in restraining the rate of CDI for vulnerable entities such as cirrhotic patients [12]. Despite the prevalence of CDI among hospitalized patients, few studies assessed the impact of CDI on patients with cirrhosis and variceal bleeding. The aim of our study is to provide an overview on patients with cirrhosis and variceal bleeding, which developed CDI after antibiotic prophylaxis.

## 2. Materials and Methods

### 2.1. Study Design

This was a retrospective, single-centre cohort study involving the assessment of information from cirrhotic patient records with or without CDI, who presented to the University County Hospital of Craiova, Romania, from 1 January 2017 to 31 December 2019. All patients signed an informed consent form at the hospital upon admission, conforming to the Declaration of Helsinki, 1967. The study was approved by the University of Medicine and Pharmacy of Craiova Ethics Commission no. 88/2020.

The patients-related variables included demographics, age, alcoholism, comorbidities, previous use of PPI, or antibiotics. Medical history was registered: cardiovascular and pulmonary diseases, diabetes mellitus, chronic renal failure, hepatocellular carcinoma (CHC). The variables related to admission were analysed: Child–Pugh score (total bilirubin, albumin, INR, ascites, encephalopathy), Atlas score (age, systemic antibiotics during CDI therapy, leukocyte count, albumin, creatinine), MELD (dialysis at least twice in the past week, creatinine, bilirubin, INR, sodium), Charlson comorbidity index (age, myocardial infarction, peripheral vascular disease, history of cerebrovascular accident, dementia, COPD, connective tissue disease, peptic ulcer disease, liver disease, diabetes mellitus, hemiplegia, chronic kidney disease, solid tumour, leukaemia, lymphoma, aids), albumin (g/dL), C-reactive protein (CRP, mg/dL), leukocytes (cells/μL), neutrophils (%), erythrocytes (cells/μL), haemoglobin (g/dL), haematocrit (%), platelet count (cells/μL), creatinine (mg/dL), urea (mg/mL), glomerular filtration rate (mL/min/1.73 m^2^), Na (mEq/L), K (mEq/L). Costs were also considered in the outcomes’ analysis.

### 2.2. Patient Admission Protocol

We only included patients ≥ 18 years old, admitted with variceal bleeding secondary to liver cirrhosis at their first episode. We followed the European Society of Gastrointestinal Endoscopy (ESGE) and American Gastroenterological Association (AGA) guidelines for variceal bleeding with immediate resuscitation, if necessary, terlipressin prescription as well as antibiotic use to prevent bacterial dissemination. Endoscopy was performed as soon as possible, pointing out either oesophageal or gastric varices. Endoscopic signs such as active bleeding, oozing and white nipple are red signs requiring immediate therapy. Band ligation was performed for oesophageal varices and sclerotherapy with n-butyl-2-cyanoacrylate for gastric varices. According to their status, patients were followed up for the next few days in the intensive care unit (ICU) or in our clinic with medical management. If rebleeding reappeared, another upper endoscopy was performed. All patients received antibiotics (ceftriaxone) according to the European Association for the Study of the Liver (EASL) recommendations [11] before or after endoscopy. Patients were also given lactulose to prevent hepatic encephalopathy.

Patients were excluded if they previously received treatment with metronidazole or vancomycin, had a recent positive test for CDI and had CDI treatment before testing. Additionally, if other antibiotics were used during their hospitalization before CDI positive results, patients were not included. Patients redirected from other hospitals that did not respect our upper gastrointestinal bleeding (UGIB) protocol were not included.

### 2.3. CDI Diagnosis

Patients developing symptoms 48 h after admission with more than 3 watery stools/per day for two consecutive days associated with abdominal pain were tested for CDI by A/B stool assay. Results were further confirmed by an enzyme immunoassay (EIA) for detecting glutamate dehydrogenase according to the European Society of Clinical Microbiology and Infectious Disease (ESCMID) [13]. We also checked the prescription records for all patients, as well as if other antibiotics were prescribed in the previous months, recent admissions for other conditions. Only one test result was used in this study. Patients received vancomycin or vancomycin +/− metronidazole depending on disease severity and therapeutic response.

### 2.4. Statistical Analysis

Continuous variables for the study sample were described as using mean and standard deviation or median (interquartile range). Percentages were used to describe the categorical variables. Comparison among groups was conducted with the Mann–Whitney U test for continuous variables and with the chi-square test for categorical variables. We created violin plots to visually compare measured parameters between two groups. Violin plots are like box plots, but also show the probability density of the data at different values. Univariate and multivariate analyses were performed to find the significant independent predictors of mortality. Variables that had a univariate association with overall mortality in CDI patients at *p*-value < 0.5 were included in the multivariate analysis. The same method was used to identify the risk factors of CDI. Correlogram with hierarchical clustering of covariates was drawn to reveal the best models for predictors of CDI. The Hosmer and Lemeshow test was used to assess how well the model fit with the data (the null hypothesis is that the model is an adequate fit). The 95% confidence interval for the odds ratio was assessed for every predictor. The area under the receiver operating characteristic (AUC) and its 95% CI was used to assess prognostic accuracy for models. A model is considered outstanding if AUC is bigger than 0.9, excellent if AUC is between 0.80 and 0.89, and acceptable if its AUC is between 0.7 and 0.8, and its 95% CI of AUC exceeds 0.7. All results were considered significant at a significance probability below 5%. All data were analysed using the GraphPad Prism 9.1.0 software (GraphPad Software, San Diego, CA, USA) and R *corrplot* and *pROC* packages (version 4.0.3).

## 3. Results

### 3.1. Patient Characteristics

A total of 367 patients with cirrhosis were identified during the study period, meeting inclusion criteria. From these patients, 25 patients were confirmed with CDI. The clinical features, comorbidities, and characteristics of patients with CDI are summarized in Table 1. The mean age of cirrhotic patients with CDI was 65.8 years (SD: 8.06 years), and 76% of the patients were male.

There were no significant differences in MELD value, the level of haemoglobin, platelets, and urea between patients with CDI and without CDI. The number of the days of admission was higher for patients with CDI (Table 2). The Child–Pugh score was significantly higher for the CDI group (*p* < 0.0001). Patients with cirrhosis and CDI had higher albumin and creatinine level than the patients with cirrhosis, but without CDI. The percentage of deaths was lower in the group of patients without CDI (*n* = 87, 25%) than in the group of patients with CDI (*n* = 11, 44%). Cirrhotic with CDI patients had a significantly higher costs (EUR 1502.45 vs. 998.31, *p*-value = 0.0006) compared with those without CDI (on an average were 1.5-fold greater). Cirrhotic patients with CDI had a higher Charlson index than cirrhotic patients without CDI (8.64 vs. 6.31, *p*-value = 0.001).

There was a modest trend for a higher rebleeding rate in patients without CDI at 7% compared with 4% for CDI patients; however, this did not reach the level of statistical significance (*p*-value = 0.53). There was a statistically significant trend for higher out-patients PPI usage in the patients with CDI at 92% compared with 48% without CDI.

**Figure 1:**
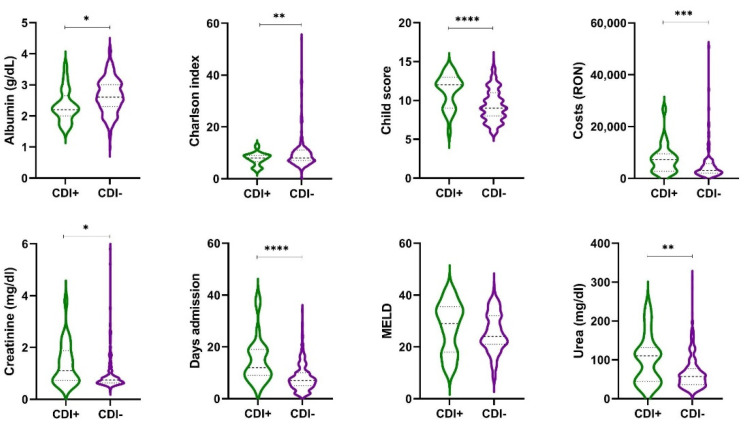
Violin plots showing the expression distribution of values of variables between Cirr+CDI+ and Cirr+CDI− patients. *, *p*-value < 0.05; **, *p*-value < 0.01; ***, *p*-value < 0.001; ****, *p*-value < 0.0001.

The comparison of the violin plots demonstrated that patients with CDI tended to have a more skewed distribution of Charlson index, Child–Pugh score, costs, creatinine, urea or admission’s days than patients without CDI, with their values toward higher values, as shown in Figure 1.

### 3.2. Factors Associated with Mortality in Cirrhotic Patients with CDI

As shown in Table 3, univariate predictors of mortality for the cirrhosis and CDI patients included Child–Pugh score (*p*-value = 0.026), leukocytes (*p*-value = 0.023), CRP (*p*-value = 0.012), Atlas score (*p*-value = 0.011) and MELD (*p*-value *=* 0.022).

According to the multivariate model, MELD is a predictor of mortality for patients with cirrhosis and CDI (Table 4). The logistic regression model was statistically significant, χ^2^(6) = 7.21, *p* = 0.032. The model explained 74.5% (Nagelkerke R^2^) of the variance in mortality and correct classified 96% of cases. Increasing MELD was associated with an increase in the likelihood of mortality.

### 3.3. Risk Factors Associated with CDI in Cirrhotic Patients with Variceal Bleeding

Table 5 summarize the results of the univariate analysis to describe the risk factors associated with CDI. Higher age (OR = 1.062; 95% CI: 1.017–1.109), higher length of hospital admission (OR = 1.115; 95% CI: 1.062–1.171), liver cancer (OR = 3.173; 95% CI: 1.245–8.087), the use of proton pump inhibitor (OR = 12.902; 95% CI: 2.995–55.566), higher level of urea (OR = 1.013; 95% CI: 1.006–1.020) and a higher Charlson index (OR = 1.609; 95% CI: 1.33–1.947) were the risk factors for CDI during the hospitalization.

Multivariate analysis showed that a higher age (OR = 1.067; 95% CI: 1.004–1.134), longer hospital stay (OR = 1.159; 95% CI: 1.086–1.238), higher level of urea (OR = 1.013; 95% CI: 1.002–1.023), higher Charlson index (OR = 1.671; 95% CI: 1.326–2.106) and the use of proton pump inhibitor (OR = 23.015; 95% CI: 4.311–52.854) were risk factors for CDI in cirrhotic patients (Table 6).

**Figure 2:**
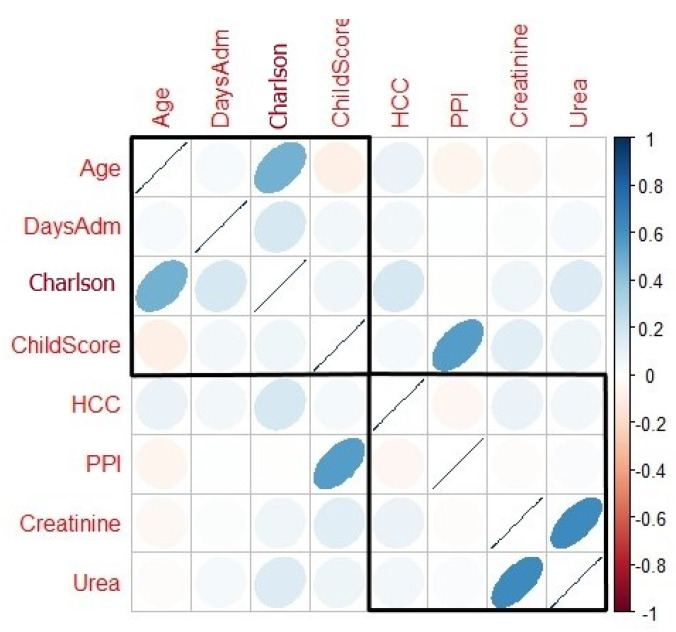
Correlogram with a hierarchical clustering of covariates included in the logistic regression analysis. Positive correlations are displayed in blue and negative correlations in red. Colour intensity and the size of the ellipse are proportional to the correlation coefficients.

The correlation values in the Spearman correlation matrix ordered by hierarchical clustering and visualized by correlogram (Figure 2) identified two clusters, which will be assessed as Model 1 (age, days of admission, Charlson index, Child–Pugh score) and Model 2 (HCC, PPI, creatinine, urea). The highest positively correlated parameters seen in the correlogram were Charlson with age (r = 0.47, *p*-value < 0.05), Child–Pugh score with PPI (r = 0.55, *p*-value < 0.05), and creatinine with urea (r = 0.64, *p*-value < 0.05).

**Figure 3:**
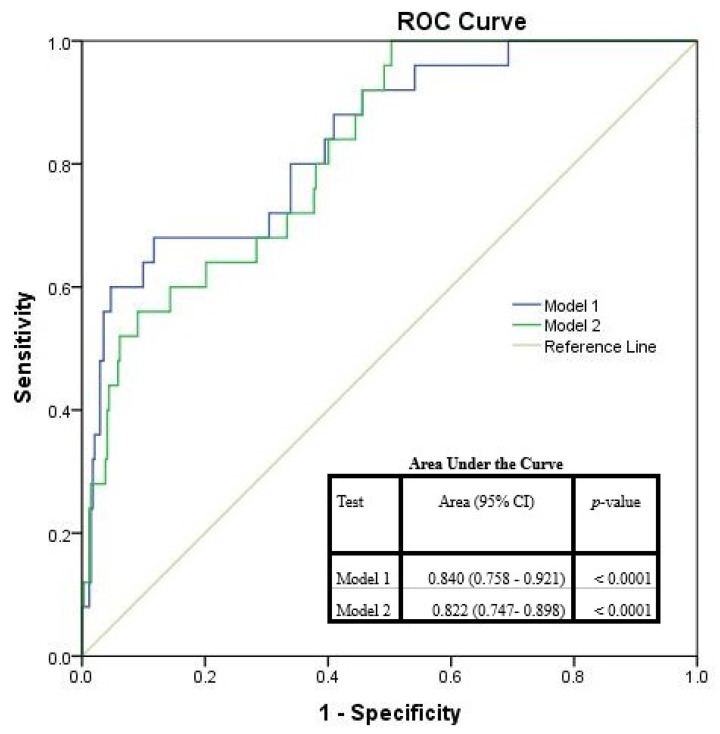
The ROC curves for Model 1 and Model 2. The ROC of a perfect predictive model has sensibility (true positive rate) equal to 1 and 1-specificity (false positive rate) equal to 0.

The accuracy of the two models, evaluated by AUC, as in Figure 3, suggested Model 1 (covariates age, days of submission, Charlson index, Child–Pugh score) being the best to predict CDI in cirrhotic patients with variceal bleeding. According to our results, the probability *p* of developing CDI after antibiotic prophylaxis could be assessed with the formula as in Equation (1):

(1) $$\log(\frac{p}{1-p})=-14.884+0.067\;\times \;\mathrm{Age}+0.122\;\times \;\mathrm{DaysAdm}+0.281\;\times \;\mathrm{Charlson}+0.463\;\times \;\mathrm{ChildScore}$$

## 4. Discussion

As the most frequent hospital-associated infection, CDI will presumably first target chronic patients, which also tend to be more susceptible to bacterial infection. However, the healthcare systems are all aware of this potential threat, as CDI should have been discussed at a rather global scale since it has also shifted to community onset [14]. The environment stands as a high-ground factor in disease evolution, namely because of asymptomatic colonization as well as spore-resistant circumstances within medical care systems, which will eventually lead to new virulent strains [15]. Thus, first focusing on a targeted group of patients who require specific antibiotics might help restrain morbidity and health costs and enable new approaches to prevent widespread infection.

Antibiotic exposure represents the highest risk for CDI, with the most common agents represented by the second and third generation of cephalosporins [16]. Depending on the patient status, even a brief exposure may be incriminated for CDI appearance. Our study focused on a specific group of cirrhotic patients who presented with variceal bleeding, pointing out a 7.20% development of CDI after ceftriaxone administration in order to prevent bacterial translocation. In addition to antibiotic prophylaxis, patients suffering from advanced chronic liver disease are at risk of acquiring nosocomial infections due to their recurrent need for hospitalizations and the additional complications they develop over time [17]. Moreover, cirrhosis may lead to an impaired local gut immune response with a low motility level which may cause bacterial overgrowth, thus hampering the CDI exposure [18].

Variceal bleeding is a major complication of cirrhosis and portal hypertension with high mortality, ranging from 15 to 20% [19]. Available guidelines suggest the use of cephalosporines for antibiotic prophylaxis for patients admitted with variceal bleeding [10,11]. Cirrhotic patients have an increased risk for bacterial infection due to increased intestinal permeability, immune dysfunction and bacterial translocation [20]. The recommendation is to use antibiotics as soon as possible, since the bacterial infection may lead to a high mortality rate. The main choice remains ceftriaxone 1 g/24 h for up to seven days followed by norfloxacin 400 mg if patients do not have advanced liver disease [11]. Antibiotic use was proved to be efficient and to decrease early bacterial infection in cirrhotic patients [21]. However, the use of antibiotics on cirrhotic patients and the potential hospital-acquired infection is not extensively discussed. Only one study analysed the CDI relevance after antibiotic administration [22]. However, the results are rather doubtable since they used metronidazole, which is not actually indicated for variceal bleeding infection risk, but also included in the CDI management.

While all antibiotics may be incriminated for hospital-related CDI, some antimicrobial agents are known to associate a higher risk than others such as fluorochinolones, cephalosporins, carbapenems and clindamycin [23]. Ceftriaxone, a third generation cephalosporine, is commonly used for bacterial prevention in patients with variceal bleeding. However, since its elimination occurs through the biliary system, the bile concentration of antibiotic will be higher within the gut, which may cause a dysbiosis in the microbiota [24].

PPIs are frequently prescribed in patients with cirrhosis, even though their indication is limited to gastroduodenal ulcers or after band ligation. Their benefit outside these causes is controversial, and their long-term use has been linked to cirrhosis complications such as spontaneous bacterial peritonitis and hepatic encephalopathy [25]. PPI influence on CDI development has been well documented. Several meta-analyses concluded that their use may foster CDI appearance, thus promoting them as an independent risk factor [26,27,28,29]. Several theories were suggested, mainly based on the PPI’s effect with acid suppression [30] and gene expression decrease, which maintain the colonocyte integrity [31] or by decreasing microbial diversity [32]. While histamine-2-receptor antagonists may be less harmful, the use of PPI along with antibiotics may enhance the risk of CDI [33]. Our study confirms that PPI might influence CDI development since patients were on a long-term therapy.

Our first objective was to highlight possible risk factors that may lead to CDI. We identified that older patients, longer admission, urea, higher Charlson index, liver cancer and PPI may influence CDI appearance. Thus, our results are similar with other studies which assessed CDI and cirrhosis. Furthermore, we tried to develop a patient profile to statistically identify a model that might help differentiate high risk patients. By hierarchical clustering, we identified two models that suggested what patients might be more predisposed to develop CDI. While Model 2 consisted of HCC, PPI, creatinine and urea, the best prediction model seemed to correspond to Model 1 which followed age, days of admission, Charlson index and the Child–Pugh score. Nevertheless, this might enhance awareness when antibiotics are used for cirrhotic patients with variceal bleeding and also help identify early additional complications. Hence, the medical system will also benefit, since this tool will help in early decision making and integrate it in sustainable public health programs through economic and environmental domains. The CDI risk tool will be helpful and might be embedded in the local protocols and decision-making process.

CDI is known to prolong admission, and consecutively raises the health costs [9]. This was also observed in our study, with patients with CDI having significantly higher costs than the ones without CDI. This provides an additional burden to the healthcare system increasing hospital charges. Bajaj et al. [34] reported that patients with cirrhosis and CDI have a 2.2-fold greater costs than those without CDI. A strategy was proposed by Saab et al. [35] who tried to implement a screening strategy to reduce healthcare costs. They concluded that patients with cirrhosis might require screening regarding their symptoms, and consecutively, the cost will be reduced at least four times compared with patients without CDI.

Mortality in CDI patients might be related to age, albumin levels, leukocytes count and renal failure [36]. Several studies identified risk factors associated with mortality in cirrhotic patients, which acquired CDI [37,38,39]. While ICU admission and albumin were recognized as predictors of short-term mortality, we identified some risk factors related to CDI severity, such as leukocytes, CRP and Atlas score, and to the liver disease—mainly Child–Pugh score and MELD. Malignancy has also been reported to increase death rate; however, our patients with HCC with associated CDI did not have a worse prognosis than other CDI patients. Hong et al. [40] obtained a hazard ratio of 1.06 ± 0.02 for the MELD score, suggesting a 21.5% increase in mortality in CDI patients and concluding that this is the only reliable short-term mortality predictor. Our analysis also recognized the MELD score as the most reliable predictor of mortality and its increase was associated with the likelihood of mortality.

A major concern for CDI exposure should now be acknowledged along with the COVID-19 pandemic. Its impact on the healthcare system should not be considered only in the short term by affecting patient’s status, medical workers [41] and the fact that some diseases might be neglected but also in the long-run by the use of specific medication and their effect on patients [42]. However, at first, the CDI rate might be on a lower trend, due to increase hand hygiene and contact precautions, but most of all due to extensive cleaning and general disinfection, along with more patients which develop severe disease, as antibiotics will be used more frequently to prevent additional bacterial pulmonary infection [43,44].

Extensive measures should be considered for dedicated patients with known risk factors when antibiotics are used. Preventing a possible CDI infection might be challenging in cirrhotic patients which require antibiotics, however, if a better knowledge of hand hygiene for both patients and medical staff is instated within hospital policies, it might prevent disease spread [45,46]. Nevertheless, isolating patients, chemical disinfection agents as well as glove use on CDI patients should be considered as infection control measures to limit other cases appearance [47].

Thus, new CDI policies on awareness should be developed and identifying a patient profile might ease day-to-day practice and decrease morbidity and healthcare costs, at least in our group of patients.

Several limitations should be considered in our study. First of all, this was a single-centre retrospective study, using a small number of CDI patients, thus, when compared to other dedicated institutions, results may differ. However, we tried to minimize potential errors by limiting to a specific set of patients and a specific antibiotic. Therapy consisted in vancomycin and vancomycin +/− metronidazole, but with no follow up in all patients for 14 days and also with no data included regarding recurrence rate. We also did not include data about CDI strains and long-term mortality. Moreover, the recurrence rate of CDI was not taken into account since we focused on the first diagnosis, risk factors and early mortality.

## 5. Conclusions

CDI infection should be considered whenever ceftriaxone is used to prevent bacterial infection after variceal bleeding and identifying the patients with a higher risk will have an impact on morbidity and on the healthcare system. Our model consisting of age, days of admission, Child–Pugh score and Charlson index could predict CDI development in cirrhotic patients with variceal bleeding following ceftriaxone. Furthermore, multicentre studies should be implemented to validate our results.

## Figures and Tables

**Table 1:** Demographics and clinical characteristics of the study population.

	Cirrhotic with CDI Patients Mean (±S.D.)/Number of Patients (%) Total *n* = 25
Age (years)	65.8 (±8.06)
Alcohol	20 (80%)
Death (yes)	11 (44%)
Time between admission and CDI diagnosis	4.16 (±1.4)
Viral	
HBV	3 (12%)
HCV	5 (20%)
No	17 (68%)
Hepatic cancer	7 (28%)
ICU	15 (60%)
Atlas score	4.32 (2.3)
PPI	23 (92%)
Rifaximin	5 (20%)
Encephalopathy	20 (80%)
Ascites	25 (100%)
SBP	5 (20%)
CRP (mg/mL)	50.4 (±23.82)
Leukocytes (cells/μL)	15,610.68 (±6900.72)
Neutrophils (%)	79.61 (±9.78)
Erythrocytes (cells/μL)	5305.44 (±6422.44)
Haematocrit (%)	27.61 (±7.67)
Glomerular filtration rate (mL/min/1.73 m^2^)	73.42 (±45.55)
Na (mEq/L)	129.96 (±4.84)
K (mEq/L)	4.45 (±0.85)

PPI, proton pump inhibitor; ICU, intensive care unit; SBP, spontaneous bacterial peritonitis; CRP, C-reactive protein.

**Table 2:** Comparison of Cirr+CDI+ and Cirr+CDI−.

Characteristics	Cirr+CDI+ *n* = 25	Cirr+CDI− *n* = 342	*p*-Value
Death (yes)	11 (44%)	89 (25.6%)	0.0446 *
Rebleeding rate	1 (4%)	23 (6.73%)	0.5321
Proton pump inhibitor (yes)	23 (92%)	164 (47.95%)	<0.0001 *
Days admission	14.84 (8.87) 12 (9–19)	8.08 (5.22) 7 (5–10)	<0.0001 *
Child–Pugh score	11.20 (2.14) 12 (9–13)	7.9 (3.83) 9 (7–10)	<0.0001 *
MELD	10.9 (15.64) 8.32 (6.54–9.25)	14.79 (69.35) 8.3 (6.66–10.5)	0.98
Albumin (g/dL)	2.33 (0.52) 2.2 (2–2.65)	2.45 (0.88) 2.6 (2.1–3)	0.013 *
Haemoglobin (g/dL)	8.73 (2.55) 8.8 (7.03–10.15)	13.7 (69.37) 8.09 (6.48–10)	0.368
Platelet count (cells/μL)	136,661.6 (111,271.56) 114,000 (80,500–168,000)	110,425.07 (77,579.62) 93,000 (64,112.5–137,000)	0.095
Creatinine (mg/dL)	1.35 (0.81) 1.1 (0.73–1.88)	0.96 (0.75) 0.74 (0.63–0.92)	0.037 *
Urea (mg/dL)	101.68 (62.79) 110 (44.5–131.5)	64.03 (42.24) 55.5 (36–77)	0.368
Costs (EUR)	1502.45 (1125.94) 1448.47 (558.99–1898.34)	998.31 (1253.4) 618.41 (412.38–1142.08)	0.0006 *
Charlson index	8.64 (3.46) 8.0 (5.50–12.00)	6.31 (1.83) 6.0 (5.0–8.0)	0.001 *

Mean (S.D.) and median (interquartile range) for continuous variables; *n* (percentage) for categorical variables; MELD, Model for End-Stage Liver Disease; Cirr+CDI+, cirrhotic with CDI patients; Cirr+CDI−, cirrhotic without CDI patients; *, *p*-value < 0.05.

**Table 3:** Univariate regression to explore factors associated with mortality.

Factors	COR	95%CI	*p*-Value
Age	1.03	0.929–1.142	0.57
Days admission	0.919	0.816–1.035	0.163
Child–Pugh score	3.787	1.174–12.208	0.026 *
Alcohol (yes)	2.25	0.304–16.632	0.427
Virala HBV HCV	0.364 1.333	0.047–2.817 0.067–26.618	0.333 0.851
Diabetes (yes)	2.0	0.366–10.919	0.423
Hepatic cancer (yes)	5.0	0.74–33.777	0.099
Rifaximin (yes)	7.429	0.69–79.957	0.098
HDS (yes)	0.205	0.018–2.327	0.201
SBP (yes)	4.875	0.43–55.292	0.201
Proton pump inhibitor (yes)	0.00	-	0.99
Albumin (g/dL)	0.167	0.021–1.315	0.089
Platelet (cells/μL)	1	1–1	0.413
Leukocytes (cells/μL)	1	1–1.001	0.023 *
CRP	1.139	1.029–1.261	0.012 *
Atlas	4.22	1.387–12.837	0.011 *
MELD	1.48	1.058–2.07	0.022 *
Charlson index	63.32	0.0–96.1	0.996

COR, crude odds ratio; PPI, proton pump inhibitor; ICU, intensive care unit; SBP, spontaneous bacterial peritonitis; MELD, model for end-stage liver disease; *, *p* < 0.05, statistically significant.

**Table 4:** Multivariate regression to explore factors associated with mortality.

Factors	AOR	95%CI	*p*-Value
Child–Pugh score	1.409	0.883–2.25	0.787
Liver cancer (yes)	14.082	0.245–89.231	0.201
Rifaximin (yes)	10.039	0.005–19.27	0.55
Leukocytes	1.0	1.0–1.001	0.147
CRP	1.016	0.032–31.933	0.993
Atlas	3.704	0.622–22.072	0.15
MELD	1.281	0.098–1.643	0.042 *

AOR, adjusted odds ratio; MELD, model for end-stage liver disease; *, *p* < 0.05, statistically significant.

**Table 5:** Univariate regression to explore factors associated with CDI.

Factors	COR	95%CI	*p*-Value
Age	1.062	1.017–1.109	0.006 *
Days admission	1.115	1.062–1.171	<0.0001 *
Child–Pugh score	1.543	1.262–1.887	<0.0001 *
Alcohol (yes)	1.5	0.52–4.3	0.451
Hepatic cancer (yes)	3.173	1.245–8.087	0.016 *
Creatinine	1.376	1.0–1.892	0.050
Urea	1.013	1.006–1.020	<0.0001 *
Proton pump inhibitor (yes)	12.902	2.996–55.566	0.001 *
Charlson index	1.609	1.330–1.947	<0.0001 *

COR, crude odds ratio; *, *p* < 0.05, statistically significant.

**Table 6:** Multivariate regression to explore factors associated with CDI.

Factors	AOR	95%CI	*p*-Value
Age	1.067	1.004–1.134	0.037 *
Days admission	1.159	1.086–1.238	<0.0001 ****
Child–Pugh score	1.224	0.916–1.636	0.171
Liver cancer (yes)	2.829	0.81–9.879	0.103
Creatinine	0.784	0.305–2.016	0.614
Urea	1.013	1.002–1.023	0.020 *
Proton pump inhibitor (yes)	23.015	4.311–52.854	<0.0001 ****
Charlson index	1.671	1.326–2.106	<0.0001 ****

AOR, adjusted odds ratio; *, *p* < 0.05; ****, *p*-value < 0.0001, statistically significant.

## Data Availability

Data supporting the results of this study are available from the corresponding author on request.
